# Expression profile of circular RNA and construction of circular RNA-Micro RNA network in salivary adenoid cystic carcinoma

**DOI:** 10.1186/s12935-020-01681-2

**Published:** 2021-01-07

**Authors:** Jing Han, Nannan Han, Zhimin Xu, Chunye Zhang, Jiannan Liu, Min Ruan

**Affiliations:** 1 Departments of Oral and Maxillofacial-Head and Neck Oncology, Ninth People’s Hospital, Shanghai Jiao Tong University School of Medicine, 639 Zhi Zao Ju Road, Shanghai, 200011 People’s Republic of China; 2 Department of Oral and Maxillofacial Surgery, School and Hospital of Stomatology, Jilin University, Changchun, 130021 China; 3 Department of Oral Pathology, Ninth People’s Hospital, Shanghai Jiao Tong University School of Medicine, Shanghai, 200011 China

**Keywords:** Circular RNA, Salivary adenoid cystic carcinoma, microRNA, RNA sequencing, circRNA ABCA13

## Abstract

**Background:**

Circular RNAs (circRNAs) is a newly discovered type of non-coding RNA, the abnormal expression of which has been demonstrated in many types of human tumors. So they have been considered as promising candidates as diagnostic and therapeutic targets in cancer. This research aimed to screen the profile of circRNA expression in salivary adenoid cystic carcinoma (SACC).

**Methods:**

Using the threshold of FDR < 0.05 and fold change > 2 or < 0.5, 5 up-regulated and 26 down-regulated circRNAs were identified. The reliability of sequencing was verified by the expression detection of randomly selected circRNAs via qRT-PCR.

**Results:**

Moreover, the circRNA-miRNA system was established by bioinformatics approaches and successfully identified an interaction between circRNA ABCA13 and a cancer-related miRNA (miR-138-5p), which was also verified by qRT-PCR. Moreover, the predicted molecular interaction proved that circRNA ABCA13 may promote SACC through inhibition of miR-138-5p.

**Conclusions:**

Collectively, this study has offered the first report about the circRNA expression profile and circRNA-miRNA network in SACC. All of the above could benefit the exploration of novel therapeutic target in SACC treatment.

## Background

Salivary adenoid cystic carcinoma (SACC) is regarded as one of the most frequently diagnosed malignant salivary gland tumors, holding 1% of all head and neck malignant tumors, and accounting for 30.42% of all malignant epithelial tumors of the salivary gland [1, 2]. Clinicopathological studies have suggested that SACC is extremely invasive, inducing distal hematogenous metastasis through blood vessel invasion, inducing perineural invasion through the infiltration of nerves. Statistics showed that the organ metastasis rate of SACC was as high as 40.9% [3, 4]. Despite some remarkable achievements have been made recently, the etiology and pathogenesis of SACC remain largely unclear [5]. Over the past years, many studies concentrated in seeking biomarkers to improve the clinical prognosis of SACC, but the results showed that current chemotherapy and molecular targeting drugs can only achieve temporary partial response or stable disease in advanced SACC with local recurrence or distant metastasis [6, 7]. Accordingly, currently the mortality of SACC still remains high and the prognosis still remains poor [3]. Therefore, a deeper understanding of SACC-related biomarkers is of great importance in revealing the pathogenesis of SACC and could provide basis for the progression of novel targeted therapies with higher efficacy.

The emergence of new generation of sequencing technology, high-throughput sequencing, is a revolutionary improvement in sequencing technology, through which more and more important genes in the tumorigenesis and development of human cancers have been screened out [8–11]. Moreover, high-throughput transcriptome sequencing provides a promising method to study the pathogenesis of SACC. It is of great significance to elucidate the molecular mechanism of SACC and find specific molecular markers for early diagnosis as well as intervention of tumor.

Up to now, the abnormal expression of various non-coding RNAs including lncRNAs and miRNAs has been demonstrated to be closely correlated to the pathogenesis of human cancers [12–14]. Circular RNA (circRNA) is one of the most recently discovered non-coding RNAs forming a closed-loop circRNA via binding between 3′ and 5′ ends [15, 16]. Researches have suggested that circRNAs could evade the degradation of nucleic acid exonucleases, thus having a high degree of stability and resistance to RNA degradation pathways, which endows them the promising potential to act as cancer biomarkers [17–20]. Previous studies have confirmed that circRNAs were differentially expressed in tumors, thereby playing the role of oncogene or tumor suppressor gene, and are closely related to biological characteristics of tumors. Moreover, circRNAs are able to inhibit of many cancer-related miRNAs expression, and the circRNA-miRNA-mRNA interaction cascade acts a regulatory role in several cancer-related pathways, which can either inhibit or promote the development and progress of cancer [21]. As far as we konw, circRNA-related studies have rarely been reported in the field of SACC, and therefore no detailed description of the relationship between circRNA-miRNA-mRNA cascade and SACC has been made. This study aimed to identify novel circRNA biomarkers by recognizing SACC-related abnormal expression of circRNA by next generation RNA sequencing analysis. The further analysis of the relationship between these circRNAs and their potential association with miRNAs could further promote our understanding of the SACC pathogenesis and propose potential therapy targets for SACC treatment.

## Methods

### Patient enrollment and tissues collection

Samples came from SACC patients who had finished the operation in the Department of Maxillofacial Head and Neck Oncology, Ninth People’s Hospital Affiliated to Medical College of Shanghai Jiaotong University from March 2016 to January 2017. People receiving any cancer treatment before admission were kept out from this study. After tumors resection, tumors and adjacent tissues were frozen in liquid nitrogen, and stored at − 80 °C. Histopathological examination as well as diagnosis of tumors were performed independently by two pathologists. Six matched SACC tissue samples and corresponding para-carcinoma tissues were examined by secondary sequencing, which were also investigated by real-time qPCR. The research was approved by the Ethics Committee of Shanghai Ninth People's Hospital and all participants in this research has signed the informed consent form.

### Sample preparation

Total RNA from cell was extracted by Trizol reagent (Invitrogen, CA, USA). The total RNA was quantified using Bioanalyzer 2200 (Agilent Technologies, CA, USA), and then stored at −  80 °C.

### cDNA library construction

The cDNA libraries were established by VAHTSTM Total RNA-seq (H/M/R). Briefly, divalent cations was used for depletion of rRNA and fragmenting into 150–200 bp at 94 °C for 8 min, and RNA fragments were then reverse-transcribed into first-strand cDNA, and second-strand cDNA was synthesized, fragments were finally end repaired VAHTSTM DNA Clean Beads were used for harvesting target bands. The products were purified and enriched by PCR which were used for creating the final cDNA libraries then quantified by Agilent2200, and then pooling the labeled cDNA libraries in equal proportion, sequenced in a single lane of the Illumina HiSeqTM 2500 with 51 plus 7 cycles by NovelBio Corp. Laboratory, Shanghai, China to generate 150 bp paired-end.

### RNA sequencing mapping

Clean reads were obtained from the original reads via expurgating the adaptor sequences before mapping, over 20% of bases with qualities of < 20 were contained by low-quality reads as well as reads with > 5% vague bases (noted as N), and the clean reads were then aligned to Human genome (version: GRCh38) through the HISAT2 program [22].

### CircRNA identification and quantification

The pipeline ‘acfs’ which was able to be used widely at https://code.google.com/p/acfs/ was used for identifying each samples’ circRNA comprising of the reported steps [23]. BOWTIE2 version 2.2.5 was used for mapping the respective reference genome [GRCH37.p13 NCBI]. Unmapped Reads were used for identifying the circRNA through BWA mem (bwa mem -t 1-k 16-T 20): local alignments of fragments in a single read that mapped to (i) regions of the same chromosome which are less than 1 Mb apart from each other (ii) on the same chain (iii) however, in the opposite order, kept them as candidates which supported junction between head to tail. Intensity of potential splice sites was then estimated, which were supported by these junction reads via MaxEntScan33, and determined the accurate juncture by choosing the donor and receptor sites with the highest splicing intensity score. Finally we reported candidate circRNAs if at least 2 reads held out the head-to-tail junction and the splicing score ≥ 10.

### Expression analysis

To evaluate circRNA expression, the BWA-mem was used for realigning all the unmapped reads to circRNA candidates under the following parameters (bwa mem-t 1-k 16-T 20). We concatenated sequences at the 5′ to 3′ end to form circular junctures, and counted the readings of each candidate which were mapped to the juncture (with at least 6 nt overhang).

### Dif-gene-finder

DESeq algorithm was utilized for filtering the differentially expressed genes, and the following are the threshold represented by FDR and *P* value analysis:

i. Fold Change > 2 or < 0.5;
ii. FDR < 0.05.

### GO analysis

The GO annotations from UniProt (http://www.uniprot.org/), the Gene Ontology (http://www.geneontology.org/) and NCBI (http://www.ncbi.nlm.nih.gov/) were used. In addition, we applied Fisher’s exact test for identifying the significant GO categories and used FDR for correcting the *P* values.

### Pathway analysis

The pathway analysis was used for looking for the differential genes’ significant pathway in accordance with KEGG database. Our group selected the significant pathway through passing the Fisher’s exact test, and made use of *P* value and FDR for defining the threshold of significance.

### GO-tree

We built GO-tree on the basis of the Gene Ontology Directed Acyclic Graph for providing user with friendly visualization and data navigation. The significant GO-Term (*P* Value < 0.01) was selected in GO Analysis in accordance with the significantly up- and down-regulated genes expression for constructing the GO-Tree in order to sum up the functions which were influenced in this assay.

### Path-act-network

KEGG was comprised of membrane transport, signal transduction, cell cycle pathways and metabolism. Genes were picked in abound biological pathway and Cytoscape was used to graphically represent the pathways, and then we made use of the KEGG database for building a genetic network based on the proteins, compounds in the database and the relationship among the genes.

### Target analysis

We utilize the Miranda as the tools for predicting differentially expressed miRNA Target on circRNA, lncRNA and mRNA.

### Real time-PCR

Total RNA was extracted via Trizol (Invitrogen, CA, USA) and Direct-zol™ RNA MiniPrep (Zymo Research, Orange, CA, USA) according to the instructions of the manufacturer, and quantified the RNA using Nanodrop 2000 (Thermo Fisher Scientific, MA, USA). We obtained first-strand cDNA by M-MLV reverse transcriptase (RT) (Promega), arbitrary primers and 1 µg of total RNA and the cDNA templates were stored at − 20 °C. The PCR products were separated by 20 g/L agarose gel, stained by ethidium bromide, observed under ultraviolet light. qPCR was performed using AceQ qPCR SYBR Green Master Mix (Vazyme). 2^−∆∆Ct^ method was used for the quantification of expressions. In this experiment, β-actin was chosen for the internal control.

### Statistical analysis

Our group expressed the data as mean ± SD (n = 6) and analyzed it by GraphPad Prism 6 software (GraphPad Software Inc., San Diego, CA, USA). In addition, t-test was used for comparing the difference. It was considered to be statistical significance that *P* value < 0.05.

## Results

### Identification of differentially expressed circRNAs

**Fig. 1:**
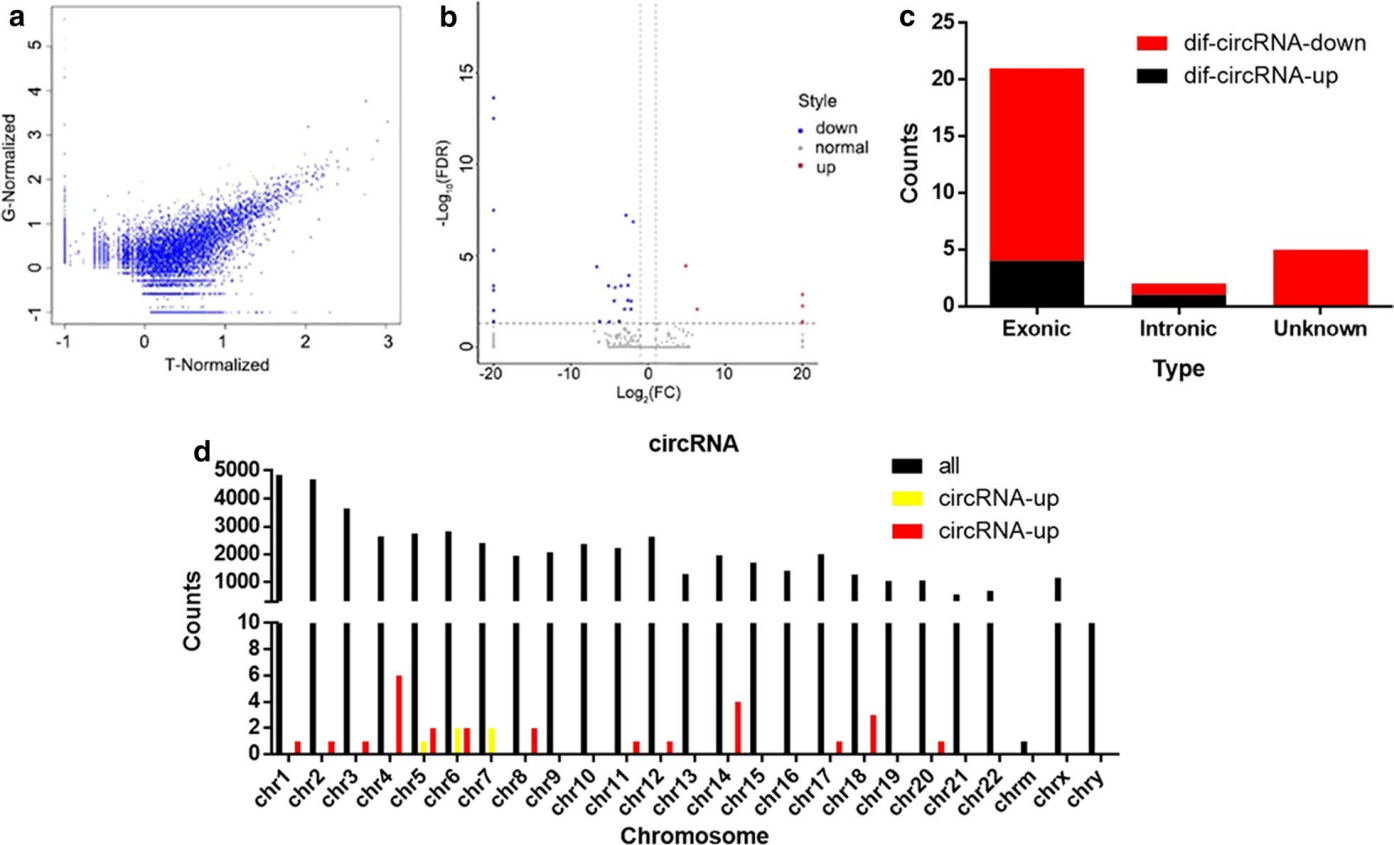
Characterizations in circRNA expression patterns of SACC and matched normal tissues. **a** The difference in the expression of circRNAs in SACC and matched normal tissues was shown by scattered plot. **b** Volcano plot shows the differential expression of circRNA between the two groups. The red dots indicated the up-regulated circRNAs, the blue dots indicated the down-regulated circRNAs. **c** The type of differentially expressed circRNAs. **d** The distribution of all circRNAs and the differentially expressed circRNAs in human chromosomes

**Fig. 2:**
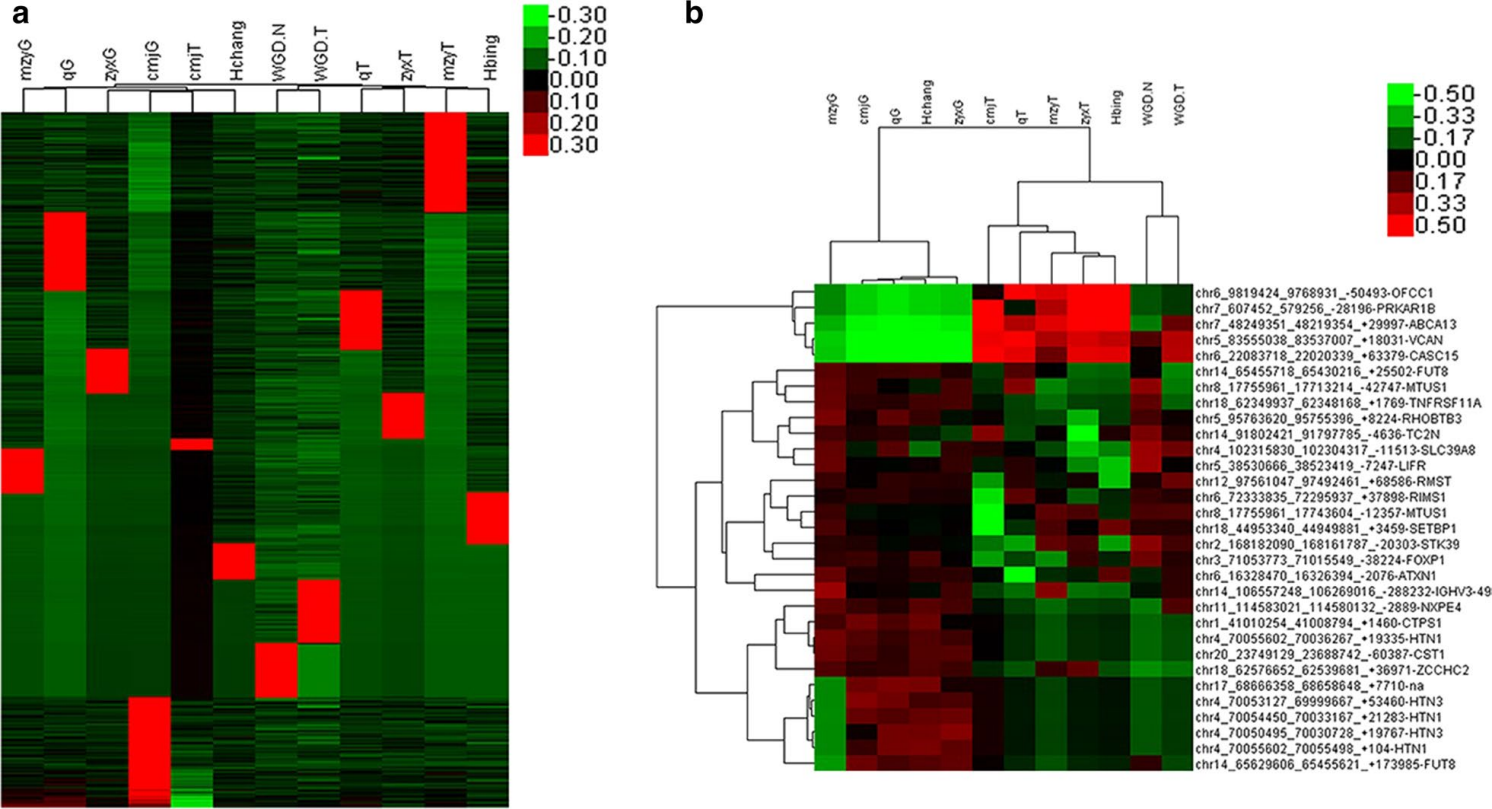
Hierarchical clusters of all circRNAs and differentially expressed circRNAs in two groups. **a** All circRNAs. **b** Differentially expressed circRNAs

Our group performed the high-throughput sequencing for evaluating circRNA expression in the SACC samples (T group) in comparison with the matched normal samples (G group). The results showed that 49475 circRNAs were detected through high-throughput sequencing. The normalized log_2_ scales were shown by the scatter plot to illustrate the differences between the two groups of samples (Fig. 1a) and we also displayed the identified circRNAs with deregulated expression in the volcano plot (Fig. 1b). In Table 1, the differentially circRNAs expression and the related information were listed. As demonstrated, 31 differentially expressed circRNAs were detected through using the fold-change filtering (absolute log_2_ (fold change) > 1, and FDR < 0.05). Compared to the matched normal tissues, 26 circRNAs were markedly down-regulated and 5 circRNAs were up-regulated in SACC tissues. Moreover, the sequencing results showed that, among the 31 differentially expressed circRNAs, 21 of them (17 down-regulated and 4 up-regulated) were covered in the exon of genomic, 2 of them (1 down-regulated and 1 up-regulated) were in the intronic genomic, while the rest were unknown (Fig. 1c). Furthermore, the chromosome distribution of the detected circRNA and differentially expressed circRNA was shown in Fig. 1d. Finally, our group utilized hierarchical clustering to illustrate the expression patterns of both all the circRNAs and the differentially circRNAs expression in the samples (Fig. 2).

**Table 1:** The differentially expressed circRNAs identified by sequencing

AccID	Log2FC	FDR	Style
chr4_70054450_70033167_+ 21283-HTN1	− 20	7.06E−19	Down
chr20_23749129_23688742_− 60387-CST1	− 20	2.37E−14	Down
chr4_70055602_70036267_ + 19335-HTN1	− 20	3.11E−13	Down
chr4_70050495_70030728_ + 19767-HTN3	− 20	3.24E−08	Down
chr5_38530666_38523419_− 7247-LIFR	− 2.85451	6.08E−08	Down
chr5_95763620_95755396_ + 8224-RHOBTB3	− 1.93144	1.40E−07	Down
chr4_70053127_69999667_ + 53460-HTN3	− 20	5.08E−06	Down
chr5_83555038_83537007_ + 18031-VCAN	4.895841	3.58E−05	Up
chr14_65455718_65430216_ + 25502-FUT8	− 6.63764	3.97E−05	Down
chr8_17755961_17743604_− 12357-MTUS1	− 2.4546	1.21E−04	Down
chr4_102315830_102304317_− 11513-SLC39A8	− 2.59543	4.19E−04	Down
chr14_65629606_65455621_ + 173985-FUT8	− 20	4.39E−04	Down
chr8_17755961_17713214_− 42747-MTUS1	− 5.11127	4.41E−04	Down
chr2_168182090_168161787_− 20303-STK39	− 3.51308	4.41E−04	Down
chr6_72333835_72295937_ + 37898-RIMS1	− 4.27145	5.54E−04	Down
chr18_62349937_62348168_ + 1769-TNFRSF11A	− 20	7.79E−04	Down
chr7_48249351_48219354_ + 29997-ABCA13	20	0.001307	Up
chr18_44953340_44949881_ + 3459-SETBP1	− 2.62237	0.002782	Down
chr12_97561047_97492461_ + 68586-RMST	− 4.38816	0.002961	Down
chr3_71053773_71015549_− 38224-FOXP1	− 2.16996	0.003033	Down
chr7_607452_579256_− 28196-PRKAR1B	20	0.005568	Up
chr14_91802421_91797785_− 4636-TC2N	− 3.04538	0.008415	Down
chr6_16328470_16326394_− 2076-ATXN1	− 2.21408	0.008415	Down
chr6_22083718_22020339_ + 63379-CASC15	6.346491	0.008415	Up
chr4_70055602_70055498_ + 104-HTN1	− 20	0.009976	Down
chr1_41010254_41008794_ + 1460-CTPS1	− 20	0.039106	Down
chr14_106557248_106269016_− 288232-IGHV3-49	− 3.72791	0.039106	Down
chr11_114583021_114580132_− 2889-NXPE4	− 6.26001	0.039492	Down
chr17_68666358_68658648_ + 7710-na	− 20	0.040683	Down
chr6_9819424_9768931_− 50493-OFCC1	20	0.040683	Up
chr18_62576652_62539681_ + 36971-ZCCHC2	− 5.02868	0.041316	Down

### Validation of the reliability of the sequencing data

**Fig. 3:**
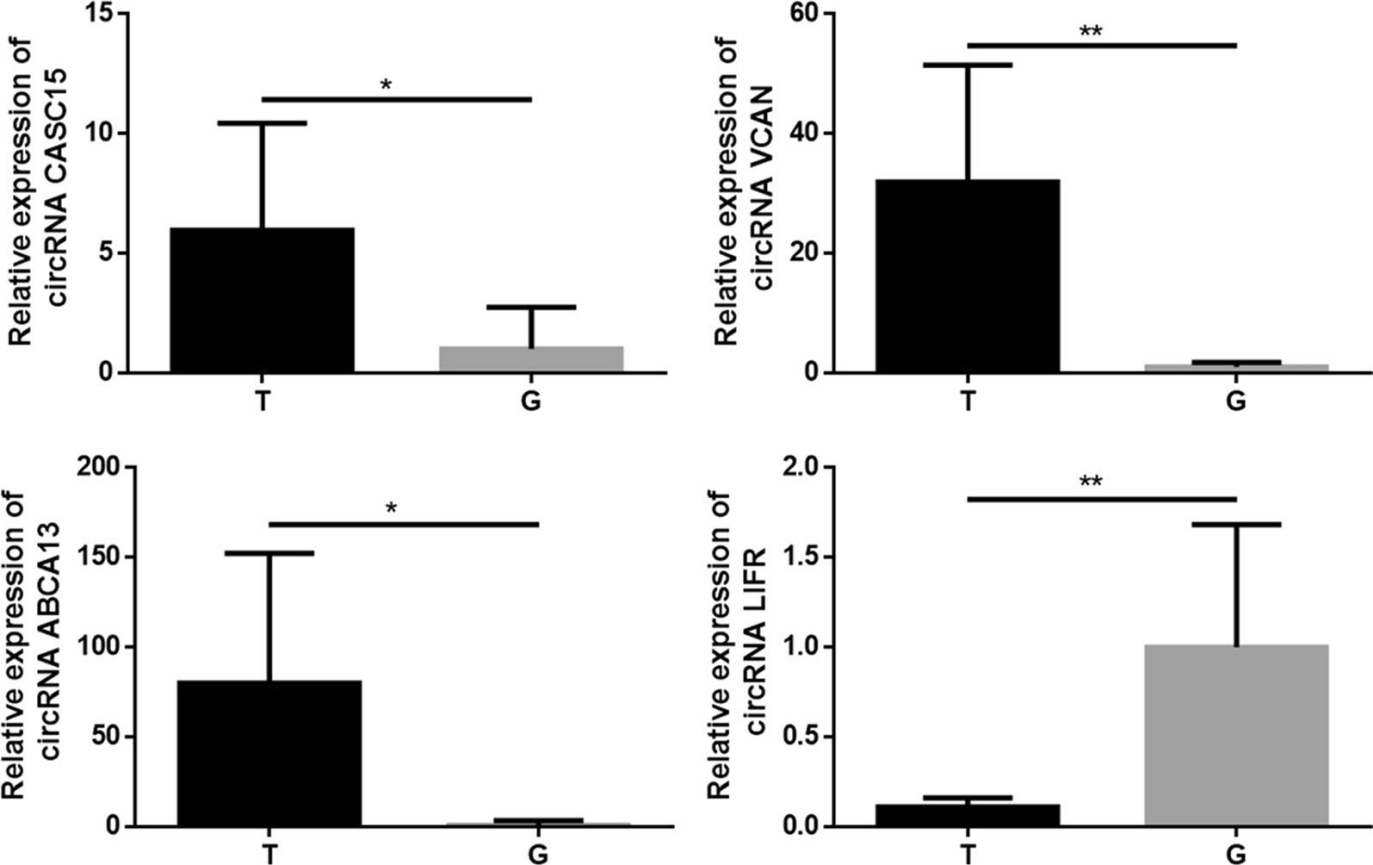
Verification of the reliability of RNA sequencing. qRT-PCR was performed for detecting the expression of circRNA circRNA ABCA13, circRNA CASC15, circRNA VCAN and circRNA LIFR in SACC and matched normal tissues. Data are shown as mean ± SD. * P < 0.05, ** P < 0.01, *** P < 0.001

For the aim to validate the reliability of the RNA sequencing, 3 up-regulated circRNA ABCA13, circRNA CASC15 and circRNA VCAN, and 1 down-regulated circRNA LIFR were selected randomly as the target of detection whose expression levels were discovered by qRT-PCR in the SACC tissues and matched normal tissues. As illustrated by Fig. 3, the qRT-PCR results were concordant with the sequencing data, indicating that the circRNA profile had high reliability.

### GO and KEGG analysis of differentially expressed circRNAs

**Fig. 4:**
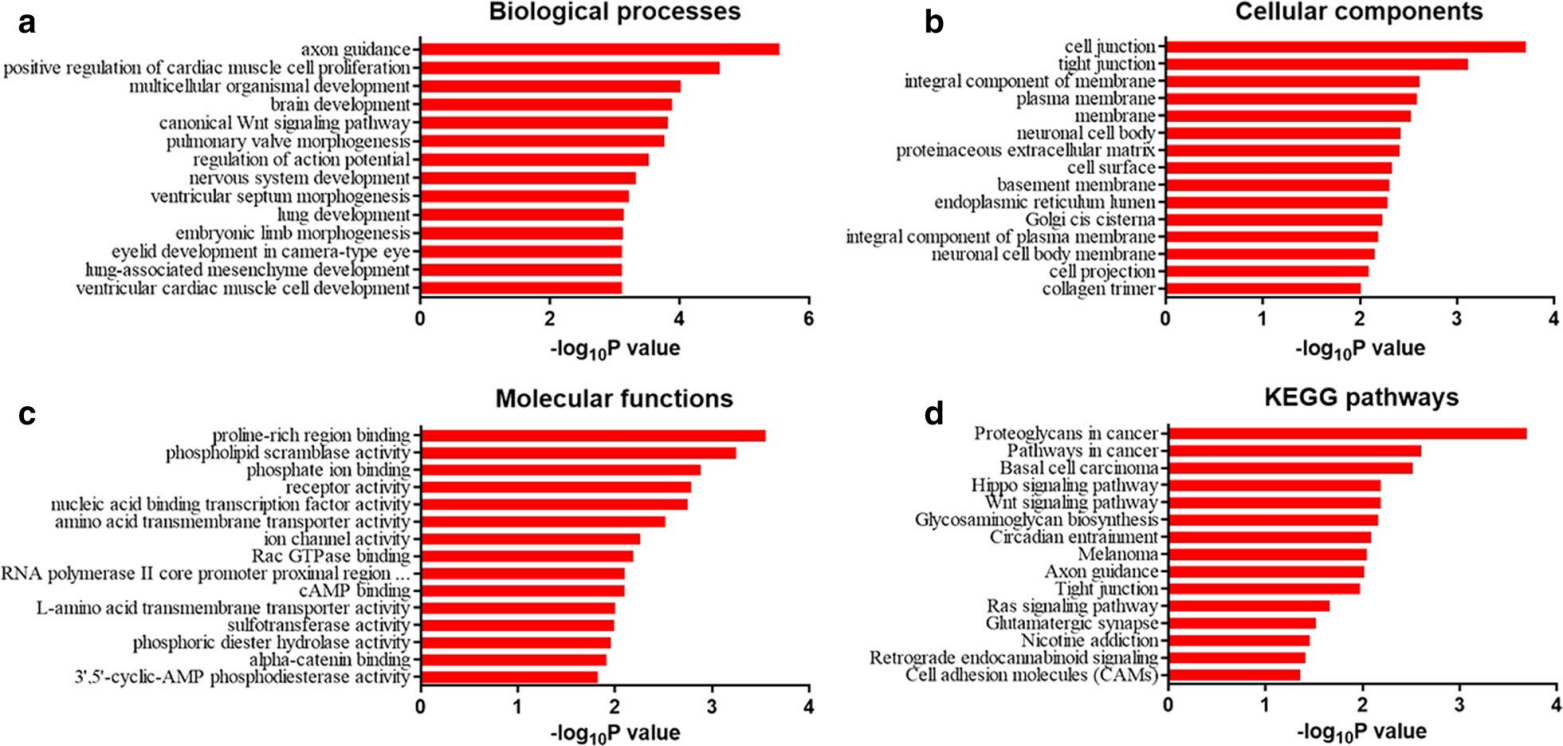
GO and KEGG pathway analysis. **a** Biological processes of GO database. **b** Cellular component of GO database. **c** Molecular functions of GO database. **d** KEGG pathway analysis

**Fig. 5:**
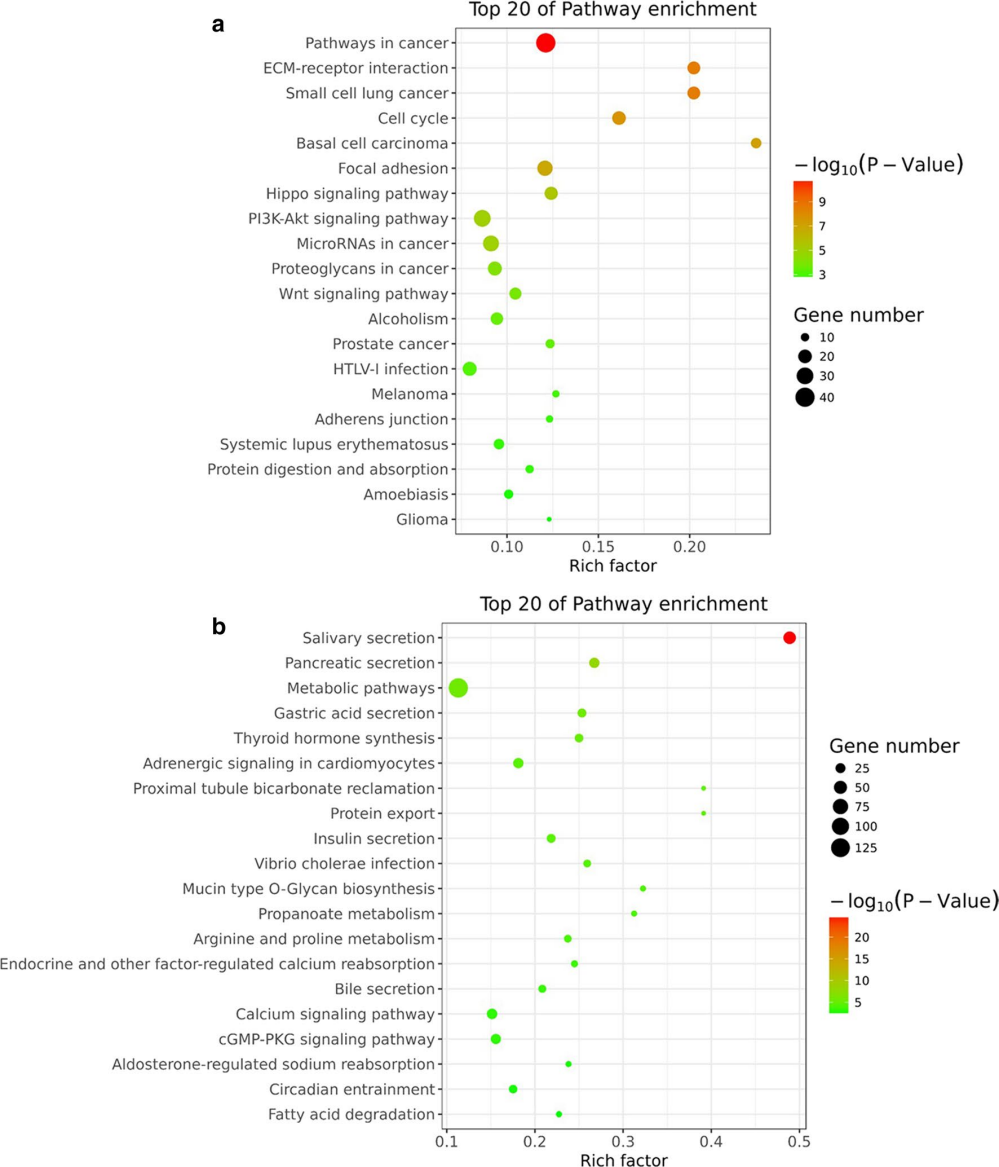
Bulb map of KEGG analysis. **a** Bulb map of KEGG analysis for up-regulated circRNAs. **b** Bulb map of KEGG analysis for down-regulated circRNAs

In order for further exploring the functionalities of the differentially expressed circRNAs, our group performed GO enrichment analysis and KEGG pathway analysis on the ceRNA-related mRNAs with the dysregulated circRNAs based on negative correlation between circRNA and miRNA, the negative correlation between the mRNA and the miRNA, and thus the positive correlation between circRNA and mRNA. As shown in Fig. 4a, GO analysis demonstrated that canonical Wnt signaling pathway, which plays important role in human cancers, and multicellular organismal development were two of the biological processes that were most likely related to the differentially expressed circRNAs. Moreover, the analysis indicated that the dysregulated circRNAs could also have impact in the molecular functions such as proline-rich region binding and phospholipid scramblase activity, and cellular components such as cell junction and tight junction (Fig. 4b, c). Subsequently, the KEGG analysis for the circRNAs showed the strong enrichment in proteoglycan in cancer and pathways in cancer such as Wnt signaling pathway and Hippo signaling pathway (Fig. 4d). Moreover, the bulb map of KEGG analysis for the up-regulated circRNAs also showed distinct enrichment in pathways in cancer and that for the down-regulated circRNAs suggested a significant enrichment in salivary secretion (Fig. 5). Taken together, the results of GO and KEGG analysis provided the potential mechanism of circRNAs in SACC.

### Establishment of the circRNA-miRNA network

**Fig. 6:**
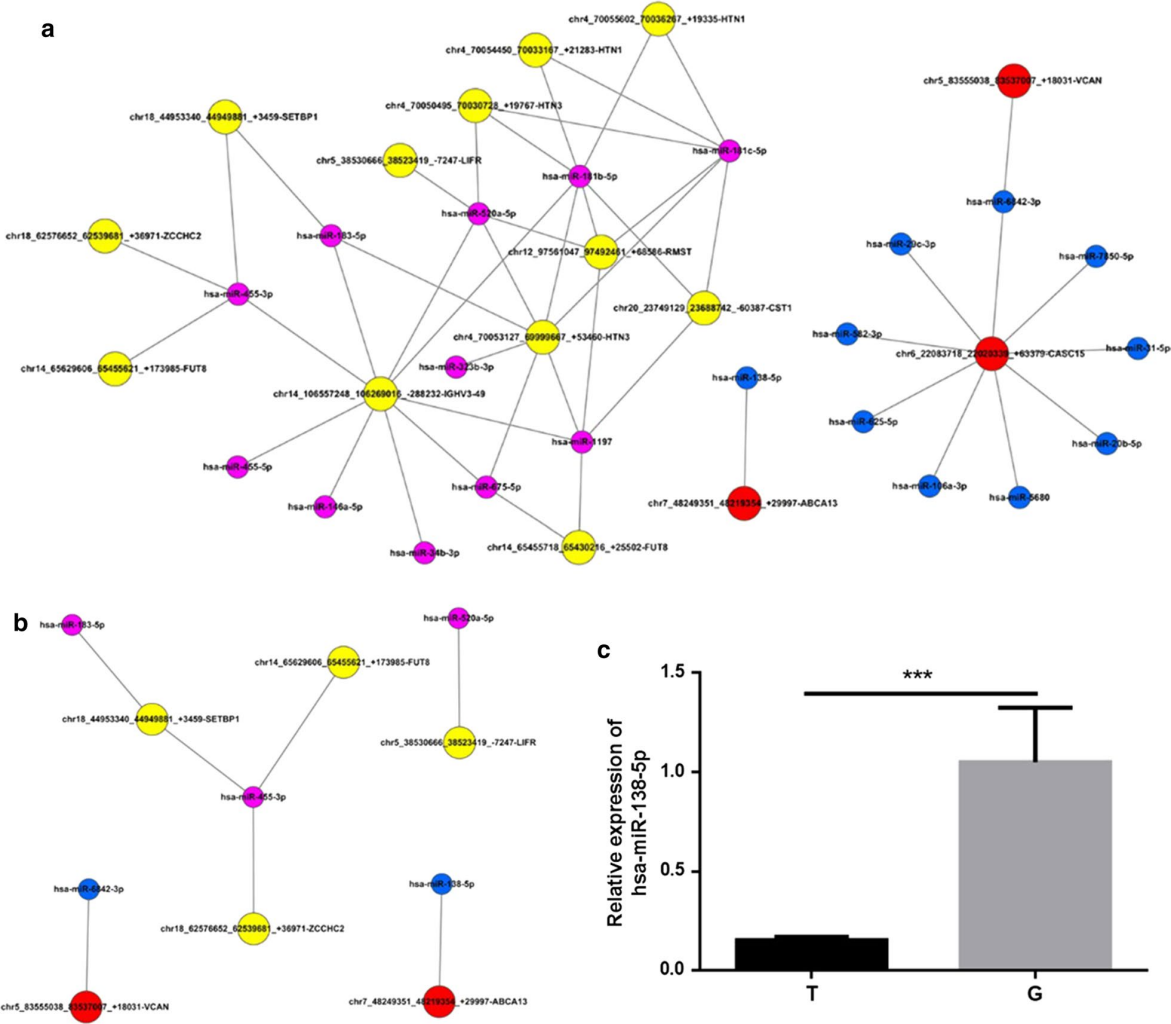
The circRNA-miRNA network. **a** The general view of circRNA-miRNA network for the differentially expressed circRNAs. **b** The circRNA-miRNA network for the exonic differentially expressed circRNAs. **c** The expression of miR-138-5p was detected by qRT-PCR in SACC and matched normal tissues. Data are shown as mean ± SD. * P < 0.05, ** P < 0.01, *** P < 0.001

**Fig. 7:**
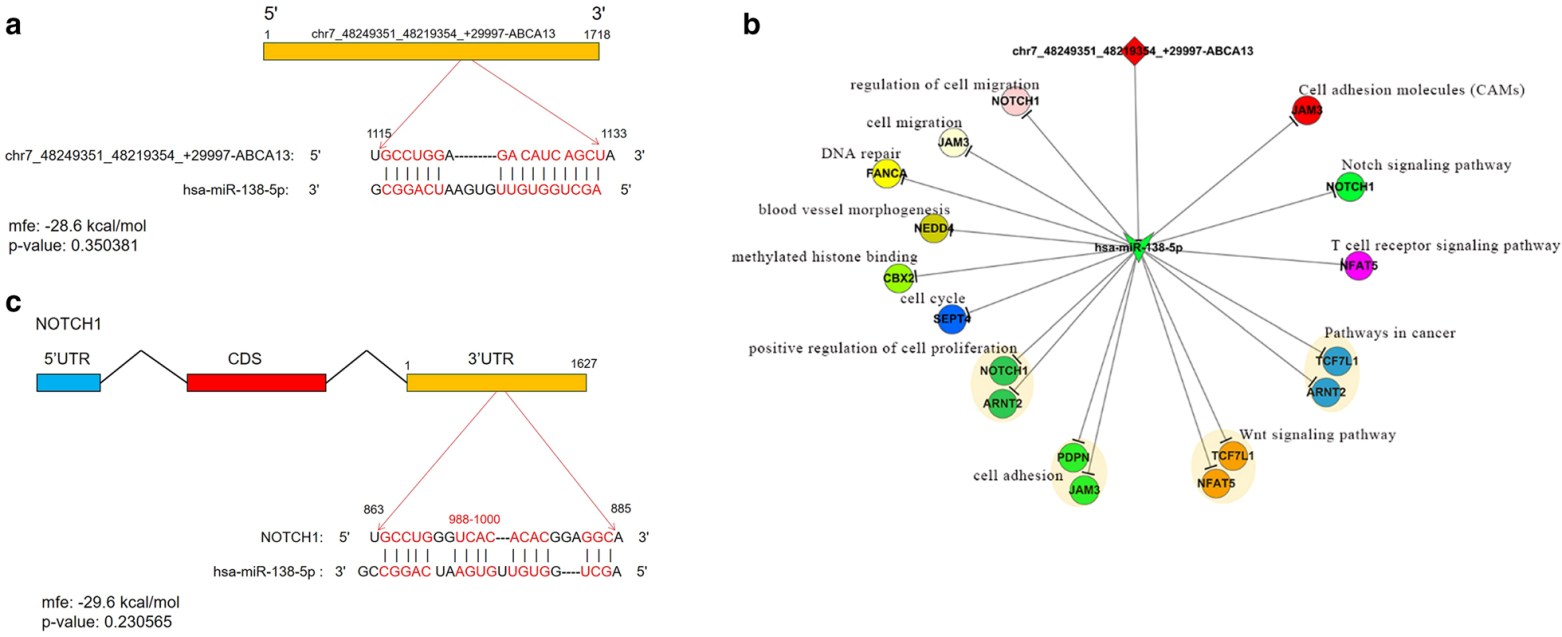
The circRNA-miRNA interaction. **a** The predicted interaction between circRNA ABCA13 and miR-138-5p. **b** The predicted ceRNA network of miR-138-5p. **c** The predicted interaction between miR-138-5p and Notch1

On the basis of the results of RNA sequencing and bioinformatics analysis, miRANDA and TargetScan databases were used for predicting miRNA targets of each conserved seed matching sequence. The results showed that there is a fairly complex regulation network between circRNA and miRNA, and Fig. 6a showed the interaction network constructed by cytoscape based on the selected pairs of circRNA-miRNA with one-to-one correspondence. Then, the interaction pairs involving exonic circRNAs were further screened and shown in Fig. 6b. Among them, we found that circRNA ABCA13 and circRNA LIFR, whose expression levels have both been verified by qRT-PCR, were predicted to interact with miR-138-5p and miR-520a-5p, respectively. In the following verification of the miRNAs by qRT-PCR, the down-regulation of miR-138-5p in SACC tissues was consistent with the predicted interaction (Fig. 6c), while the simultaneously up-regulated expression of circRNA LIFR and miR-520a-5p was not in accordance with the prediction (data not shown). The predicted molecular interaction between circRNA ABCA13 and miR-138-5p was displayed in Fig. 7a. Moreover, we also predict the regulation network of miR-138-5p and showed the molecular interaction between miR-138-5p and Notch1, which has been reported to have important role in SACC (Fig. 7b, c).

## Discussion

CircRNA, a novel kind of non-coding RNA with regulatory ability, is different from traditional mRNA and was first discovered in RNA viruses in the 1970s [15]. As the high-throughput RNA sequence technology and bioinformatics further developed, researchers have found that circRNA is ubiquitous in various species and has important physiological functions [15, 24]. Moreover, previous results have manifested that some circRNAs may play crucial parts in the development and progression of human cancers [25]. Sequencing analysis of the expression profiles of circRNA in tumor tissues and the matched adjacent tissues showed that abnormal circRNAs expression was common in tumors [25]. For example, Lu et al*.* identified 1155 differentially expressed circRNAs through the microarray detection of human breast cancer tissues and corresponding para-carcinoma tissues. They further verified that hsa_circ_100219 was differentially expressed in breast cancer tissues and normal tissues, and of important value in the diagnosis of breast cancer [26]. Another research showed that hsa_circ_0001649 expression was inhibited in hepatocellular carcinoma and positively related to the growth of tumor. Subsequent study indicated that it could regulate various genes expression, thus participating in the incidence and metastasis of hepatocellular carcinoma [27]. Moreover, researchers found a great amount of complete and stable circRNAs in human serum exosomes, and identified the significantly different expression profile of circRNAs in serum of cancer patents and normal people [18]. Collectively, all the results indicated that aberrant circRNAs expression may be taken part in the incidence and development of various human cancer.

Current studies have confirmed that miRNAs are related to almost all cellular functions and play a crucial role in the development of diseases [28]. CircRNA has abundant microRNA (miRNA) response element (MRE), which plays the role of miRNA sponge in cells, and can eliminate the regulation of miRNA on its target genes [29]. Thus, circRNAs may involve in the physiological regulation and human diseases through the interaction with miRNA. Because of the post-transcriptional regulatory effect of circRNA, circRNA has become an ideal therapeutic target, which can be used in the development of new drugs through the simulation of its molecular characteristics, providing new means for treating diseases [30]. However, the circRNA research in oncology is still in its initial stage and the relationship between circRNA and SACC is rarely reported.

In this research, we present the expression profile of circRNA in SACC for the first time by using secondary sequencing, attempting to further understand the molecular mechanism of SACC. Based on the results of RNA sequencing, it revealed that 31 circRNAs were dysregulated in SACC tissues compared to the matched normal tissues based on the filtration by the absolute of log_2_ fold change > 1 and FDR < 0.05. Among them, 26 circRNAs were significantly down-regulated and 5 circRNAs were significantly up-regulated. 4 differentially expressed circRNAs were randomly chosen for verifying the dependability of the sequencing data through detecting their expression levels in the tissue samples by qRT-PCR. The results of qRT-PCR were in perfectly consistent with the RNA sequencing data, clearly suggesting the validation of RNA sequencing.

Considering that accumulating evidence has proved that circRNA was able to regulate their parent genes expression [4], we performed GO and KEGG pathway analysis for evaluating the function of circRNAs in SACC. GO analysis revealed that axon guidance, proline-rich region binding and cell junction were the functions that are most likely regulated by the differentially expressed circRNAs among biological processes (BP), molecular functions (MF) and cellular components (CC), respectively. Notably, the Wnt signaling pathway, which has been proved to take part in various human cancers, was also potentially regulated by the differentially expressed circRNAs. On the other hand, the KEGG analysis indicated the relationship between the dysregulated circRNAs and several tumor-associated signaling pathways such as Wnt pathway, Hippo pathway and Ras pathway. Moreover, the bulb map of the up-regulated circRNAs showed the most potential involvement of pathways in cancer and that for the down-regulated circRNAs was salivary secretion, indicating their potential regulation mechanism of SACC.

In this study, we also constructed the circRNA-miRNA interaction network in SACC for the first time to further investigate the functional roles of circRNAs. Through screening the circRNA-miRNA interaction pair with one-to-one correspondence, we found that miR-138-5p and miR-520a-5p were the targets of circRNA ABCA13 and circRNA LIFR, respectively. The verification by qRT-PCR also proved the down-regulation of miR-138-5p expression in SACC tissues, which was in accordance with the negative regulation effect between circRNA and miRNA. Notably, miR-138-5p was recently reported to be able to act as a tumor inhibitor in colorectal cancer through targeting PD-L1 [31]. Therefore, circRNA ABCA13 may promote the development and progression of SACC through the inhibition of miR-138-5p expression. Interestingly, the molecular interaction analysis demonstrated not only the interaction between circRNA ABCA13 and miR-138-5p, but also the interaction between miR-138-5p and Notch1, which was recently suggested to play a critical role in the cell growth, anti-apoptosis and metastasis of SACC [32]. Despite of all the results, the detailed regulation effect and underlying mechanism of circRNA ABCA13/miR-138-5p axis still need further investigation and would be our focus in the future work.

## Conclusion

In conclusion, this study illustrated the first circRNA signature of SACC. Through the high throughput transcriptome sequencing and bioinformatics analysis, we recognized the differentially expressed circRNAs, analyzed the potential functional roles of them and constructed the circRNA-miRNA interaction network. Moreover, we demonstrated that circRNA ABCA13 may promote the development and progression of SACC through the inhibition of miR-138-5p expression. Our findings benefit for the exploration of novel therapeutic target and provide potential diagnostic markers for SACC treatment.

## Data Availability

The datasets during and/or analysed during the current study available from the corresponding author on reasonable request.
